# Data relating to change in locus of control orientation of parents overtime (from pre-birth to 20 years later), participating in the Avon Longitudinal Study of Parents and Children (ALSPAC)

**DOI:** 10.1016/j.dib.2018.07.014

**Published:** 2018-07-10

**Authors:** Yasmin Iles-Caven, Jean Golding, Genette Ellis, Steven Gregory, Stephen Nowicki

**Affiliations:** a Centre for Academic Child Health, Population Health Sciences, Bristol Medical School, University of Bristol, UK; b Department of Psychology, Emory University, Atlanta, USA

**Keywords:** ALSPAC, Prenatal parental locus of control, Psychology, Change over time, Personality

## Abstract

Locus of control (LOC) measures an individual׳s expectancy regarding their ability to affect what happens to them based on their behavior. Those with an internal LOC (ILOC) believe their own behavior influences what happens to them. Those with an external LOC (ELOC) perceive that what happens to them is beyond their control (i.e. determined by luck, fate, chance or powerful others) [1]. A vast amount of research (mainly cross-sectional) suggests that an ELOC is associated with many adverse personal, social, academic and health outcomes.

LOC data were uniquely collected *prenatally* from over 12,000 pregnant women and their partners enrolled in the Avon Longitudinal Study of Parents and Children (ALSPAC). The LOC measure used was a shortened version of the adult version of the Nowicki-Strickland Internal-External locus of control scale. This was administered to the mothers within self-completion questionnaires at three-time points: during pregnancy, at 6 and at 18 years post-partum. In parallel, self-completion questionnaires containing the same LOC questions were completed by their partners during pregnancy, at 6 and 20 years later.

ALSPAC LOC data are unique in that they measured orientation over time and on a much larger sample of respondents than is usual. We describe the scale used, why it was chosen and how individual scores changed over time.

**Specifications Table**

**Table:** 

Subject area	Psychology
More specific subject area	Locus of Control
Type of data	Tables
How data was acquired	Longitudinal cohort study questionnaires
Data format	Edited and analyzed
Experimental factors	Self-completion questionnaires
Experimental features	Item responses and mean LOC scores over time
Data source location	Former Avon area, centered around Bristol, UK
Data accessibility	Data are within this article.

**Value of the data**

• The ALSPAC dataset contains information on a large sample of women and their partners in a geographically defined population whose lives were monitored over many years.
• The data provide a basis for identification of the antecedents and consequences of possessing a particular locus of control in parents.
• The data allow detailed analyses of events and social circumstances over the lifespan and their association with changes in locus of control orientation.
• The data allow detailed analyses of the impact of parental locus of control on child and adolescent health and development.

## 1 Data

In this paper, we describe adult data on locus of control for men and women over time.

The ALSPAC study website contains details of all the data that are available through a fully searchable data dictionary: 〈http://www.bris.ac.uk/alspac/researchers/our-data/〉. Data can be obtained by bona fide researchers, for a fee, after application to the ALSPAC Executive Committee. Details on how to apply and costs involved are available on the following: (http://www.bristol.ac.uk/alspac/researchers/access/).

## 2 Experimental design, materials and methods

### 2.1 Why is locus of control (LOC) important?

Julian Rotter published the concept of locus of control (LOC) in 1966, and provided a scale with which to measure it. He defined LOC as a generalized problem-solving expectancy [1].

An internal LOC (ILOC) was found to be associated with achievement in many aspects of life and therefore may be related to success in, for example: academia [2], [3], [4]; business [5], [6]; sporting achievement [7], [8], [9]; as well as being an indicator of psychological adjustment [10], [11], [12]; and physical health [13], [14], [15]. An external locus of control (ELOC) in one or both parents can impact negatively on child behavior outcomes and the child׳s own LOC [e.g. [16], [17], [18]]. During the last 30 years, the LOC of the general US population appears to have become more external [19] but the reasons for this trend are unknown.

### 2.2 The Avon Longitudinal Study of Parents and Children (ALSPAC)

Data collections in ALSPAC were designed to determine the environmental and genetic factors that are associated with the health and development of the study offspring [20], [21], [22]. As part of the design there was a concerted effort before the child׳s birth to obtain from the parents baseline details of their personalities, moods and attitudes, including LOC. ALSPAC recruited 14,541 pregnant women resident in Avon, UK with expected dates of delivery between 1st April 1991 and 31st December 1992 (an estimated 80% of the eligible population). Of these initial pregnancies, there was a total of 14,676 fetuses, resulting in 14,062 live births, 13,988 of whom were alive at 1 year of age. Data were collected at various time-points via self-completion questionnaires, biological samples, hands-on measurements, and linkage to other data sets. For full details of all the data collected see the study website: http://www.bristol.ac.uk/alspac/researchers/our-data/. Ethical approval for the study was obtained from the ALSPAC Ethics and Law Committee [ALEC; IRB00003312] (registered on the Office of Human Research Protections database as UBristol IRB #1) and the Local Research Ethics Committees [23].

The mothers have been followed throughout the life of the study child, but partners were only included in the study with the permission of mothers (i.e. initially they were not enrolled in their own right). Mothers were given a questionnaire which they could hand to their partner if they wished; partners were given their own reply-paid envelope in which to return their completed questionnaires to avoid potential bias and protect the individual׳s confidentiality.

The three main reasons for loss to follow-up were: when the child died; the mother refused; or moved away and proved untraceable. As with all longitudinal studies, attrition rates increased over time [21], [22].

### 2.3 The LOC measure used

The LOC measure used was a shortened version of the Adult Nowicki-Strickland Internal-External locus of control scale (ANSIE) [24]. It was developed by Stephen Nowicki specifically for the ALSPAC study, comprising 12 questions taken from the original 40 question scale. The ANSIE was chosen over health-related scales because it was a generalized scale consistent with Rotter׳s definition and would have the potential to relate to a wider range of factors. This version was validated on a sample of 135 pregnant women in the US prior to use in ALSPAC. The 12-question scale (Table 1) was administered to the mothers within self-completion questionnaires at three time points: during pregnancy, and at 6 and 18 years post-partum. In parallel, during pregnancy and at 6 years post-delivery, the mothers were sent self-completion questionnaires for the partners which contained identical LOC questions. Around 20 years after the study child׳s birth, the partners were invited to a clinic, and they responded to a computerized questionnaire which included identical LOC questions. ELOC was defined as a score greater than the median and ILOC as equal to, or less than, the median.

**Table 1:** Questions used in the shortened version of the AINSIE that made up the ALSPAC locus of control score.

**Q no.**	**Questions**	**Y**	**N**
1.	Did getting good marks at school mean a great deal to you?	0	1
2.	Are you often blamed for things that just aren׳t your fault?	1	0
3.	Do you feel that most of the time it doesn’t pay to try hard because things never turn out right anyway?	1	0
4.	Do you feel that if things start out well in the morning that it׳s going to be a good day no matter what you do?	1	0
5.	Do you believe that whether or not people like you depends on how you act?	0	1
6.	Do you believe that when bad things are going to happen they are just going to happen no matter what you try to do to stop them?	1	0
7.	Do you feel that when good things happen they happen because of hard work?	0	1
8.	Do you feel that when someone doesn’t like you there׳s little you can do about it?	1	0
9.	Did you usually feel that it was almost useless to try in school because most other children were cleverer than you?	1	0
10.	Are you the kind of person who believes that planning ahead makes things turn out better?	0	1
11.	Most of the time, do you feel that you have little to say about what your family decides to do?	1	0
12.	Do you think it׳s better to be clever than to be lucky?	0	1

To create the score, the following questions were coded: Yes=1; No=0: 2, 3, 4, 6, 8, 9,11.

The remaining questions were coded as Yes=0; No=1: 1, 5, 7, 10, 12.

The responses were then summed. An ELOC was defined as a score greater than the median.

### 2.4 Responses over time associated with socio-economic factors

For the mothers, the availability of LOC data varied over time in regard to various social, economic and lifestyle features (Table 2a), with a proportional reduction in residents of publicly owned housing, young mothers, partners’ manual social class, low levels of maternal education, and smoking as measured in pregnancy. Similar biases were present in their partners (Table 2b); it should be noted that the group answering the LOC questions in the clinic (20 years post-delivery) were also different as they were, of necessity, still resident in or near the study area. The women who answered all three LOC questionnaires were at the more advantaged end of the social spectrum. Nevertheless parents from disadvantaged backgrounds are included in sufficient numbers for sub-group analyses when required.

**Table 2a:** Availability of women׳s locus of control (LOC) data at three time points, showing distributions with background factors.

**Features measured in pregnancy**	**LOC in pregnancy**	**LOC at 6 years**	**LOC at 18 years**	**LOC at all 3 time points**
Housing tenure				
Owned/mortgaged	75.2%	81.1%	86.3%	87.0%
Publicly owned	13.0%	9.2%	5.3%	5.0%
Other	11.8%	9.7%	8.4%	8.0%
*n*	12,068	8308	4043	3814
Age of mother at conception				
<20	5.5%	3.2%	1.7%	1.3%
20–24	21.2%	18.0%	14.4%	13.7%
25–34	65.5%	71.1%	74.2%	74.0%
35+	7.8%	8.7%	10.7%	11.0%
n	12,448	8526	4129	3868
Social class (partner׳s occupation)				
Non-manual	56.4%	59.5%	66.1%	66.7%
Manual	43.6%	40.5%	33.9%	33.3%
*n*	10,484	7578	3844	3614
Maternal education level				
<O-level	28.9%	23.8%	15.9%	15.2%
O-level	35.0%	35.7%	34.3%	34.2%
>O-level	36.1%	40.5%	49.8%	50.6%
*n*	11,712	8291	4072	3838
Mother was smoking mid-pregnancy				
Yes	18.8%	16.0%	11.0%	10.8%
No	81.2%	84.0%	89.0%	89.2%
*n*	12,146	8394	4080	3849

**Table 2b:** Availability of men׳s locus of control (LOC) data at three time points, showing distributions with background factors.

**Features measured in pregnancy**	**LOC in pregnancy**	**LOC at 6 years**	**LOC at 20 years**	**LOC at all 3 time points**
Housing tenure				
Owned/mortgaged	78.5%	85.0%	90.9%	91.7%
Publicly owned	11.0%	6.5%	2.7%	2.5%
Other	10.5%	8.5%	6.4%	5.8%
*n*	8404	4372	1841	1242
Age of partner at conception				
<20	4.6%	1.9%	0.6%	0.5%
20–24	20.4%	15.6%	11.0%	12.9%
25–34	67.2%	73.9%	76.3%	60.0%
35+	7.8%	9.6%	12.1%	26.6%
n	8621	4458	1876	1085
Social class (partner׳s occupation)				
Non-manual	59.5%	66.9%	74.0%	77.6%
Manual	40.5%	33.1%	26.0%	22.4%
*n*	7663	4105	1750	1201
Partner education level				
<O-level	26.6%	19.4%	12.9%	12.7%
O-level	35.2%	33.7%	31.2%	19.8%
>O-level	38.2%	47.9%	55.9%	67.5%
*n*	8243	4362	1843	1219
Partner was smoking mid-pregnancy				
Yes	16.6%	12.1%	7.3%	15.6%
No	83.4%	87.9%	92.7%	84.4%
*n*	8466	4412	1855	1215

### 2.5 Responses to LOC questions

1. The responses to the 12 questions used to create the LOC score are shown at three-time points in Table 3. It is important to note that the data shown are subject to the changes in response rates over time. For a true assessment of the individual׳s trends over time it is important to confine the analyses to individuals who answered at all three time points. In general, an examination of the statistics for the LOC scale show that women have a higher score (i.e. more external) than the partners, whether one examines the means or the medians.

**Table 3:** Frequencies of positive responses to questions in the LOC scale by mothers in pregnancy and their partners (questions where the answer ‘yes’ denotes externality are marked with an asterisk).

**Question No.**	**Mother in pregnancy**	**Mother 6** **y later**	**Mother 18** **y later**	**Partner in pregnancy**	**Partner 6** **y later**	**Partner 20** **y later**
1	68.9%	72.0%	70.0%	56.7%	65.0%	63.0%
2*	22.5%	17.1%	14.9%	21.4%	16.0%	11.8%
3*	14.1%	11.1%	6.7%	12.6%	9.4%	4.3%
4*	45.4%	44.6%	42.7%	38.1%	36.7%	39.5%
5	68.1%	73.2%	76.8%	70.7%	74.1%	81.%
6*	57.0%	52.5%	47.9%	42.8%	38.6%	33.0%
7	43.7%	51.0%	54.5%	54.4%	55.9%	61.7%
8*	58.4%	58.5%	55.6%	50.4%	50.7%	46.8%
9*	9.4%	9.1%	10.9%	9.0%	6.9%	6.6%
10	68.3%	74.6%	84.1%	74.1%	83.4%	91.6%
11*	20.2%	9.4%	10.0%	17.8%	10.3%	11.6%
12	44.8%	54.6%	57.3%	61.0%	67.0%	73.0%
**No Replied**	**12,471**	**8531**	**4175**	**8645**	**4462**	**2036**
Mean [SD]	4.37 [2.12]	3.82 [1.99]	3.52 [2.01]	3.59 [2.30]	3.29 [2.01]	2.87 [1.88]
Median	4	3	3	3	3	2

LOC=locus of control; SD=standard deviation.
2. Examination of the subgroup of women who answered the external questions at all three time points showed more reliable patterns (Table 4) – only two of the external questions showed a difference over time: Q9 increased as the women got older, and Q11 which was at its lowest when the child was 6 – presumably when the mother felt she was running the household – but increased again when the child was in late adolescence. Trends of increasing internality were apparent for Q5, Q7, Q10 and Q12.

**Table 4:** Frequencies of positive responses to questions in the LOC scale by mothers in pregnancy and their partners (questions where the answer ‘yes’ denotes externality are marked with an asterisk).

**Question No.**	**Mother in pregnancy**	**Mother 6 yr later**	**Mother 18 yr later**	**Partner in pregnancy**	**Partner 6 yr later**	**Partner 20 yr later**
1	76.94%	77.90%	69.98%	66.14%	71.00%	64.74%
2*	14.35%	14.17%	14.45%	11.95%	12.51%	11.05%
3*	7.29%	7.68%	6.10%	5.42%	5.66%	3.76%
4*	42.01%	41.73%	41.88%	27.89%	31.87%	36.43%
5	70.27%	74.59%	75.78%	74.26%	77.05%	82.26%
6*	46.87%	45.01%	46.48%	28.29%	29.32%	31.07%
7	42.04%	52.59%	52.74%	50.44%	57.05%	62.06%
8*	53.77%	56.62%	56.20%	44.22%	44.54%	45.14%
9*	6.31%	7.70%	10.47%	3.59%	3.82%	6.64%
10	76.06%	79.76%	83.43%	81.99%	88.45%	92.13%
11*	15.77%	7.91%	9.57%	11.79%	7.41%	10.73%
12	45.22%	55.92%	54.47%	61.91%	69.24%	74.06%
No. Replied	3868	3868	3868	1225	1225	1225
Mean [SD]	3.73 [1.93]	3.40 [1.88]	3.49 [1.99]	2.72 [1.93]	3.28 [2.06]	2.81 [1.86]
Median	4	3	3	2	3	3

LOC=locus of control; SD=standard deviation.
3. For the women׳s partners, there was a gradual increase in externality as measured by Q4, but the increase in positive answers to Q9 was similar to that for the women (Table 4). Trends of increasing internality were again apparent for Q5, Q7, Q10 and Q12.
4. The trends concerning the mean LOC values indicate little change in orientation in the mothers over time, but for the partners, there was an increase in externality when the children were aged six (Table 4).

### 2.6 Correlations over time and between partners

LOC measures were available for the three-time points for 3829 women. The correlations between the pregnancy measure and that 6 years later was 0.546; that between pregnancy and 18 years later was 0.535; and the correlation between the measures at 6 and 18 years was 0.559. All were highly significant (*P*<0.0001). For the men over time, fewer were involved (1244), but the correlation coefficients were very similar (0.548, 0.535 and 0.551).

Although only 959 pairs of the men and women had completed questionnaires at each of the three-time points, the results are clear (Table 5): the LOCs of the women is poorly correlated with the LOCs of their partners (*r*<0.225).

**Table 5:** Correlation between LOC measures of partnerships (*n*=959).

	**Mother in pregnancy**	**Mother 6 yr later**	**Mother 18 yr later**
Partner in pregnancy	0.223	0.142	0.159
Partner+6 years	0.176	0.154	0.145
Partner+20 years	0.186	0.146	0.158

### 2.7 Publications

To date there have been ten publications using ALSPAC data on parental LOC, summarized in Table 6, and include both analyses identifying antecedent factors [25], [26], [27], [28] and consequences [18], [29], [30], [31], [32], [33].

**Table 6:** Publications using parental locus of control in ALSPAC.

**Authors**	**Outcomes**	**Environment**	**Results**
Golding et al. [25]	LOC as measured in pregnancy	Maternal and paternal backgrounds; features of early childhood (<6 yrs)	Women׳s ELOC was associated with nine characteristics of their parental backgrounds and early childhood.
Golding et al. [26]	LOC as measured in pregnancy	Maternal and paternal backgrounds; features of mid-childhood and adolescence; traumatic events in childhood; lifestyle choices (<17 yrs)	Used exposome approach with ELOC as the outcome by hypothesis-free structured backwards stepwise logistic regression analyses. Women׳s ELOC associated with six characteristics of parental backgrounds and childhood including onset of regular smoking in mid-childhood (6–11 years) by the mother herself. Protective factors also identified.
Nowicki et al. [18]	Parental attitudes and behavior; temper tantrums; eating and sleeping behavior	Prenatal parental LOC. Compared families where both parents ILOC or ELOC or one of each	Prenatal parent ELOC is associated with the consistent maternal report of more negative child behavior outcomes.
Golding et al. [29]	Child IQ measured at 4 years using WPPSI^a^ & 8 years using WISC^b^.	Prenatal maternal LOC. Perinatal life-style exposures, parenting attitudes & strategies and socio-economic circumstances are predicted by internality and explain mechanism through which maternal ILOC influences cognition of child.	Prenatal parental ILOC is associated with higher child IQ at ages 4 (*n*=986). At 8 years (*n*=6801) the IQ was increased by as much as 7 points if the mother was ILOC.
Lekfuangfu et al. [31]	Intergenerational implications for early childhood skill formation.	Maternal LOC as measured in pregnancy. Parenting style and investment in early childhood human capital.	Maternal LOC strongly predicts attitudes towards parenting styles and time investment in their child.
Gutman et al. [30]	Nurturing parenting capability from infancy to early childhood.	Maternal LOC as measured in pregnancy. Individual characteristics of mothers and children; social networks, SES and marital relations of mother; prediction of parenting behaviors types.	Maternal ILOC had positive benefits for parenting (higher levels of educational communication) regardless of family income. Those with lower incomes fared much worse if they had an ELOC.
Golding et al. [27]	LOC as measured in pregnancy	Paternal backgrounds; features of childhood	Identical methodology to Golding et al. 2017a above. Men had many of the same antecedent characteristics as the women Associations with having ELOC: their mother׳s year of birth and father׳s social group, prenatal exposure to cigarette smoke, starting to smoke regularly <11 years, and having a friend die.
Golding et al. [33]	DXA scans of fat mass in adolescence	Prenatal parental LOC	Parent and child externality associated with greater fat mass. Factors associated with parental behavior (e.g. smoking in pregnancy, failure to breast feed and early introduction of solids) accounted for a third of the excess fat mass associated with maternal externality.
	Mean fat mass and obesity	Child LOC at age 8	
Nowicki et al. [28]	Prenatal LOC and	Stressful life events	Results suggest substantial variation within spousal dyads and moderate stability over time. Main causes of change in LOC (towards externality) were stress in relationships with partners, friends, family, financial stability, job security and illness.
	Parental LOC 6 years later		
Nowicki et al. [32]	Prenatal parental LOC	Teacher rated SDQ measured in School Years 3 and 6 by the class teacher	Greatest risk of negative behavior if both parents external, least risk if both internal. The pattern of associations varied depending on whether mother or father external, type of adverse behavior and school year. The only consistent relationship was maternal prenatal ELOC and emotional difficulties in the children.
	Goodman׳s Strengths & Difficulties Q (5 sub-scales & total)		

a WPPSI Wechsler Pre-school and Primary Scale of Intelligence.

b WISC Wechsler Intelligence Scale for Children.
